# Extracellular vesicles with altered tetraspanin CD9 and CD151 levels confer increased prostate cell motility and invasion

**DOI:** 10.1038/s41598-018-27180-z

**Published:** 2018-06-11

**Authors:** Joshua S. Brzozowski, Danielle R. Bond, Helen Jankowski, Belinda J. Goldie, Rachel Burchell, Crystal Naudin, Nathan D. Smith, Christopher J. Scarlett, Martin R. Larsen, Matthew D. Dun, Kathryn A. Skelding, Judith Weidenhofer

**Affiliations:** 1 Cancer Research Program, Hunter Medical Research Institute, New Lambton, NSW Australia; 2 School of Biomedical Sciences and Pharmacy, The University of Newcastle, Callaghan, NSW Australia; 3 School of Environmental and Life Sciences, The University of Newcastle, Ourimbah, NSW Australia; 4 Department of Biochemistry and Molecular Biology, Monash Biomedicine Discovery Institute, Monash University, Clayton, VIC Australia; 5 Emory University, Atlanta, Georgia USA; 6 ABRF, Research Services, University of Newcastle, Callaghan, NSW Australia; 7 Department of Biochemistry and Molecular Biology, University of Southern Denmark, Odense, Denmark

## Abstract

To facilitate intercellular communication, cells release nano-sized, extracellular vesicles (EVs) to transfer biological cargo to both local and distant sites. EVs are enriched in tetraspanins, two of which (CD9 and CD151) have altered expression patterns in many solid tumours, including prostate cancer, as they advance toward metastasis. We aimed to determine whether EVs from prostate cells with altered CD9 and CD151 expression could influence cellular behaviour and increase the metastatic capabilities of non-tumourigenic prostate cells. EVs were isolated by ultrafiltration and characterised for their tetraspanin expression and size distribution. iTRAQ was used to identify differences between RWPE1 and tetraspanin-modified RWPE1 EV proteomes, showing an enrichment in protein degradation pathways. Addition of EVs from RWPE1 cells with reduced CD9 or increased CD151 abundance resulted in increased invasion of RWPE1 cells, and increased migration in the case of high CD151 abundance. We have been able to show that alteration of CD9 and CD151 on prostate cells alters the proteome of their resultant EVs, and that these EVs can enhance the migratory and invasive capabilities of a non-tumourigenic prostate cellular population. This work suggests that cellular tetraspanin levels can alter EVs, potentially acting as a driver of metastasis in prostate cancer.

## Introduction

The tetraspanin superfamily is a highly conserved family of proteins with at least 33 members identified in humans, including CD9, CD63, CD81 and CD151^1^. They are involved in the regulation of a number of cellular functions, including cellular motility and migration, and as such, have demonstrated involvement in the dissemination and metastasis of tumours (reviewed by Zöller^1^). Although tetraspanins alone have not shown any intrinsic signalling pathway activations, they serve as molecular organizers of the plasma membrane of cells and facilitate the actions of their partner molecules, including integrins, members of the immunoglobulin superfamily, and matrix metalloproteinases. Tetraspanins along with their partner molecules can form tetraspanin-enriched microdomains or the ‘tetraspanin web’ that act as signalling platforms allowing tetraspanins to influence cellular functions. In prostate cancer, the altered expression of the tetraspanins CD9 and CD151 is commonly seen as a tumour progresses towards a metastatic phenotype. In these cases, CD9 expression is typically decreased and CD151 expression is typically increased, and both have been identified as having prognostic significance in prostate cancer^2–5^.

The dissemination and metastasis of a tumour is a multifactorial process involving the degradation of connective tissue, cellular migration and invasion into and back out of the circulation and lymph systems, and the resumption of proliferation within a premetastatic niche at a distant site of the body^6^. The formation of the premetastatic niche involves modulation of the extracellular matrix of a distant organ to a more favourable environment for a metastasizing tumour cell to adhere and form a secondary tumour mass^7^. This process requires a variety of different molecular drivers, including proteases that degrade matrix components and chemokines that can recruit bone marrow progenitor cells to promote angiogenesis^8^. Whilst it is not fully known why the activity and expression of these molecules changes, extracellular vesicles (EVs) have been proposed to be involved.

One of the more highly researched classes of EVs are exosomes – nano-sized membranous vesicles ranging from 30–120 nm in diameter. They are formed intraluminally, utilizing endosomal complex required for transport (ESCRT)-dependent or -independent pathways. The tetraspanin CD63 has been reported to coordinate both ESCRT-dependent and -independent pathways for exosome formation^9^, following the identification of the secretion of exosomes from cells lacking the ESCRT proteins^10^. The importance of tetraspanins in the formation and function of exosomes was further demonstrated when it was shown that dendritic cells derived from CD9 knockout mice produced fewer exosomes compared to control mice^11^, and that Tspan8 expression on exosomes was able to contribute to target cell selection^12^. There are many reported roles for exosomes in the body including antigen presentation and immune regulation^13–15^, the maintenance of homeostasis in neighbouring cells^16^ and the formation of the premetastatic niche^17,18^.

Typically, when prostate cancers become more aggressive and progress towards a metastatic phenotype, they experience alterations in tetraspanin expression, where CD9 levels decrease and CD151 levels increase^2,4^. Additionally CD9 and CD151 have been shown to form heterodimers to a small extent^19^ and therefore an integral component of the study described herein was the expression of these tetraspanins on EVs, and how these EVs can impact the function of a non-tumourigenic cellular population. EVs are increasingly being investigated for their role in the communication between cells, both locally and throughout the body. Whilst it is known that tumour cells displaying an altered CD9 and CD151 expression pattern have a higher invasive capacity than other cells, little is known as to what alteration of these tetraspanins does to the function and proteomic composition of EVs and what impact the changes will have on key metastatic functions. It is also unknown what impact these EVs will have on surrounding cells, and whether they can alter the phenotype of a non-tumourigenic cellular population to adopt a more aggressive and invasive one. In this study, we explored the function of CD9 and CD151 on prostate EVs following treatment of non-tumourigenic RWPE1 prostate cells with EVs from RWPE1 cells with altered CD9 and CD151 expression or EVs from a tumourigenic prostate cell line. We further went on to quantitatively identify differences in the proteome of these EVs to determine whether the recruitment and abundance of proteins could explain the effects observed.

## Results and Discussion

### EVs reflect the CD9 and CD151 patterns of their cells of origin

**Figure 1:**
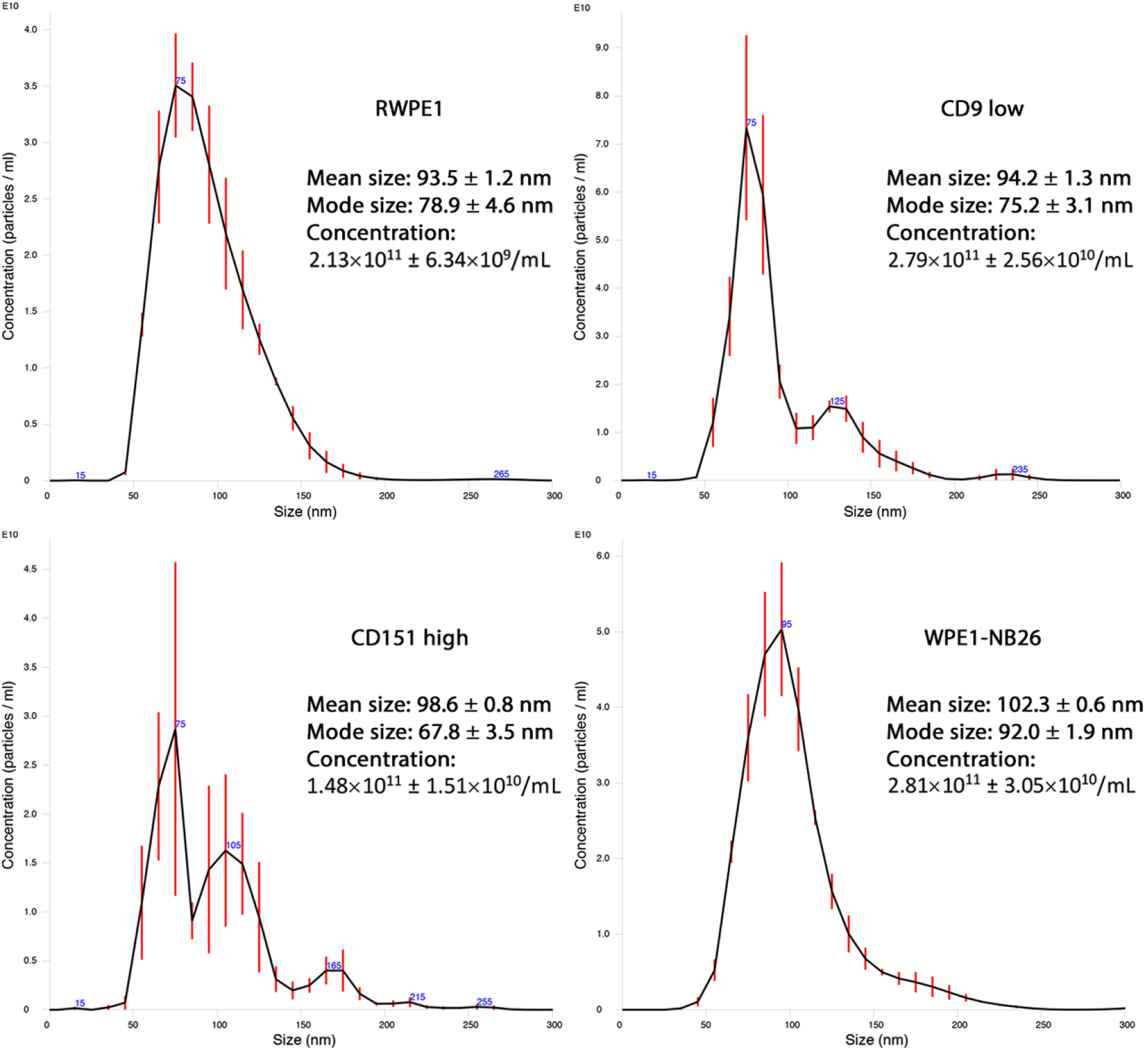
Average size distribution curve of EVs determined by NTA. Three 60 s videos were recorded for each sample, and NTA analysis settings kept constant between samples. The average concentration of vesicles was plotted against their size with error bars representing the standard deviation in concentration in 10 nm size increments. The mean and mode sizes and the average concentration are given ± standard deviation.

**Figure 2:**
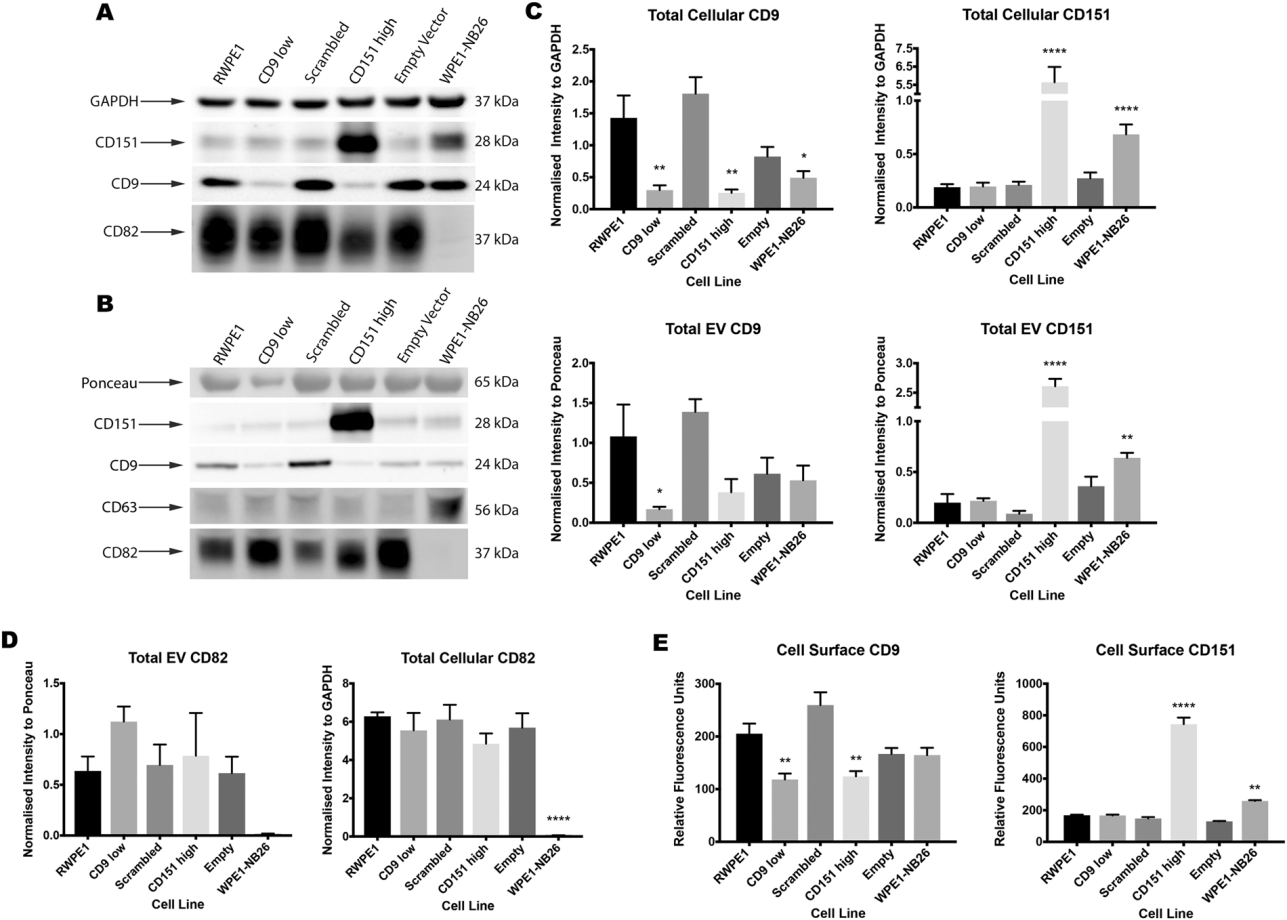
Cell and EV Tetraspanin Expression. (**A**) Cells were assessed for their total CD9, CD151 and CD82 content via western blot, with GAPDH used as a loading control. (**B**) EVs were assessed for their total CD9, CD151 and CD82 content via western blot, using Ponceau-S stained bands as a loading control. EVs were further assessed for the expression of CD63, a common EV marker. (**C**) Differences in CD9 and CD151 expression compared to RWPE1 cells and EVs were determined using one-way ANOVA with Bonferroni’s multiple comparisons test. CD9 low, CD151 high and WPE1-NB26 cells all showed a significant decrease in CD9 expression compared to RWPE1 cells, however only CD9 low EVs had a significant decrease in expression compared to RWPE1 EVs. Both CD151 high and WPE1-NB26 cells and EVs showed significant increases in CD151 expression compared to RWPE1 cells and EVs. **(D)** Differences in CD82 expression in cell and EV lysates were determined as above. WPE1-NB26 cells and EVs were negative for CD82 expression, whereas alterations in CD9 and CD151 expression did not significantly affect CD82 abundance in either cells or EVs. (**E**) Cell surface expression of CD9 and CD151 was determined using flow cytometry, and one-way ANOVA with Bonferroni’s multiple comparisons test was used to determine significance. CD9 low and CD151 high cells showed significant decreases in CD9 surface expression to RWPE1 cells. CD151 high and WPE1-NB26 cells showed significant increases in CD151 surface expression to RWPE1 cells. Data shown are representative of at least three independent experiments. *P < 0.05; **P < 0.01; ****P < 0.0001. Error bars are SEM. Western blot images are cropped, with full-length blots presented in Supplementary Fig. S7.

Considering the relationship between decreased CD9 and increased CD151 expression in prostate cancer cells with the progression towards metastasis, and the potential for EVs to drive the formation of the premetastatic niche, we were interested to see if the abundance of CD9 and CD151 on EVs was directly related to their cellular expression. Thus, we modified the expression of CD9 and CD151 in RWPE1 cells and isolated EVs from cell culture media by ultrafiltration. Characterising the EVs with nanoparticle tracking analysis (NTA) showed that altering the cellular tetraspanin profile did not alter the size of EVs, which were all within the classically defined 30–120 nm range for exosomes. As it has previously been shown that tumour cells secrete larger numbers of EVs compared to non-tumourigenic cells^20,21^, we were interested to see if altering the tetraspanin expression in RWPE1 cells altered the number of EVs produced by these cells. There were no differences in concentrations of EVs released from CD9 low or CD151 high RWPE1 cells compared to unmodified RWPE1 cells, however, the concentration of EVs was significantly decreased in CD151 high EVs compared to CD9 low (p = 0.0159) and WPE1-NB26 (p = 0.0148) EVs (Fig. 1). Further, all EVs were positive for CD9 and CD63 expression, common markers of EV populations (Fig. 2B), although differences in abundance of these markers were detected.

CD9 and CD151 abundance in cells and EVs was determined by western blot, with densitometry highlighting a significant decrease in cellular CD9 content for CD9 low (−79.14%, p = 0.0021), CD151 high (−82.10%, p = 0.0015) and WPE1-NB26 (−65.61%, p = 0.0123) cells compared to RWPE1 cells, with no significant differences in cells transfected with the Scrambled (p = 0.9162) and Empty Vector controls (p = 0.1937) (Fig. 2A,C). For CD151 expression of cells, the CD151 high cell line displayed a significant increase in CD151 protein expression compared to RWPE1 cells (2,879.85%, p < 0.0001) (Fig. 2C), however the extreme increase in CD151 expression in the CD151 high cell line was masking other significant differences between the other cell lines used when compared using Bonferroni’s multiple comparisons test. To overcome this, the CD151 high cell line was excluded from a second multiple comparisons test, and by doing so, the WPE1-NB26 cell line also displayed a significant increase in CD151 expression compared to RWPE1 cells (261.32%, p < 0.0001). CD9 low, Scrambled and Empty Vector cells all showed no significant difference in CD151 protein expression compared to RWPE1 cells (p > 0.9999).

In regards to EV tetraspanin expression, only the EVs derived from CD9 low cells displayed significantly reduced CD9 expression compared to EVs derived from RWPE1 cells (−84.27%, p = 0.0351) (Fig. 2B,C). Scrambled (p > 0.9999), CD151 high (p = 0.1644), Empty Vector (p = 0.7210) and WPE1-NB26 (p = 0.4393) EVs had no significant differences in CD9 expression compared to RWPE1 EVs. For CD151 expression, EVs derived from CD151 high (1,219.24%, p < 0.0001) and the WPE1-NB26 (223.41%, p = 0.0079) cells displayed significantly increased CD151 expression compared to EVs derived from RWPE1 cells. EVs from CD9 low (p > 0.9999), Scrambled (p > 0.9999) and Empty Vector (p = 0.8099) cells had no significant differences in CD151 expression compared to RWPE1 EVs. Further, analysing the relative abundance of CD151 and CD9 in the cells compared to the EVs showed no difference in either tetraspanin, except for CD151 in CD151 high cells and EVs (p < 0.0001), as determined by two-way ANOVA with Bonferroni’s multiple comparisons after normalising to RWPE1 abundance (data not shown). These results suggest that the abundance of CD9 and CD151 on EVs is, to a large extent, driven by the cellular abundance of the parent cell. This has potential implications for the assessment of EVs using the presence of CD9 as a marker of EV sub-populations.

To determine if the findings of altered tetraspanin abundance were restricted to CD151 and CD9 an additional tetraspanin, CD82, was also assessed in cell and EV lysates (Fig. 2A,B). CD82 functions as a metastasis suppressor by interfering with the activation of integrin β1 (ITGB1), causing a decrease in cellular motility and invasion^22^ and by stabilising E-cadherin/β-catenin complexes, maintaining strong cell-cell adhesion to inhibit the dissemination of cancer cells^23^. When compared to RWPE1 cells, CD9 low (p > 0.9999), Scrambled (p > 0.9999), CD151 high (p = 0.6410) and Empty Vector (p > 0.9999) cells did not significantly differ in CD82 expression, however, WPE1-NB26 cells had negligible CD82 expression (p < 0.0001) (Fig. 2D), indicative of the invasive and metastatic nature of this cell line. In EVs, CD82 abundance was not significantly different in CD9 low (p = 0.7073), Scrambled (p > 0.9999), CD151 high (p > 0.9999) or Empty Vector (p > 0.9999) samples compared to RWPE1 EVs (Fig. 2D). Although WPE1-NB26 EVs had negligible CD82 expression, as was seen in the cells, the difference failed to reach significance when compared to RWPE1 EVs (p = 0.3343), possibly due to the larger variation in abundance of CD82 in the EVs tested. To assess whether there were differences in the abundance of CD82 between cells and EVs, cellular and EV abundance was normalised to RWPE1 abundance and compared using two-way ANOVA with Bonferroni’s multiple comparisons. There were no significant differences observed between the cellular and EV abundance of CD82 (data not shown). Although not directly assessed here, the finding that these three tetraspanins (CD9, CD151 & CD82) show a correlation between cellular and EV expression, suggests that the other commonly used EV markers, CD63 and CD81^24^, may also show this pattern. Therefore, if these tetraspanins have low abundance in a cellular population, the expression may be undetectable in EVs, confounding the identification of EV subtypes.

Tetraspanins are known to be important in cell signalling, particularly through the organisation of the tetraspanin web on the cell surface. In order to determine whether the cell surface tetraspanin expression is responsible for the expression seen in EVs, we assessed cell surface-specific expression of CD9 and CD151 using flow cytometry. This showed the same overall pattern of CD9 expression as for the total expression with a significant decrease in CD9 low (−42.36%, p = 0.0016) and CD151 high (−39.54%, p = 0.0036) cell lines compared to the RWPE1 cell line, with no significant differences observed for the Scrambled (p = 0.0976), Empty Vector (p = 0.4636) and WPE1-NB26 (p = 0.3794) cell lines (Fig. 2E). In concordance to total CD151 expression, both the CD151 high (342.80%, p < 0.0001) and WPE1-NB26 (53.92%, p = 0.0030) cell lines displayed significantly higher surface expression of CD151 when compared to the RWPE1 cell line, with no significant differences observed for the CD9 low (p > 0.9999), Scrambled (p > 0.9999) and Empty Vector (p = 0.6250) cell lines (Fig. 2E). It has been previously shown that the binding of different monoclonal antibodies (mAb) to CD151, including mAb 11B1 used in this study, can be affected by partner molecule binding to CD151 masking the antibody binding epitope^25^. The consequence of such interactions could lead to an underrepresentation of the total CD151 content in flow cytometry experiments, where these interactions are not disrupted by detergents, as occurs during western blotting. Considering this limitation, the data here show that cellular abundance of CD9 and CD151 is directly related to abundance in EVs. Further analysis of tetraspanin abundance is required, using antibodies that recognise specific pools of bound and unbound tetraspanins, to determine if cell surface abundance is correlated with EV abundance in the same way. There have been speculations that members of the tetraspanin family are important for the formation and cargo recruitment of EVs^26^, however it is relatively unknown whether alterations in the tetraspanin expression of EVs will affect their function and composition.

### Alterations in CD9 and CD151 abundance on EVs affects their proteome

**Figure 3:**
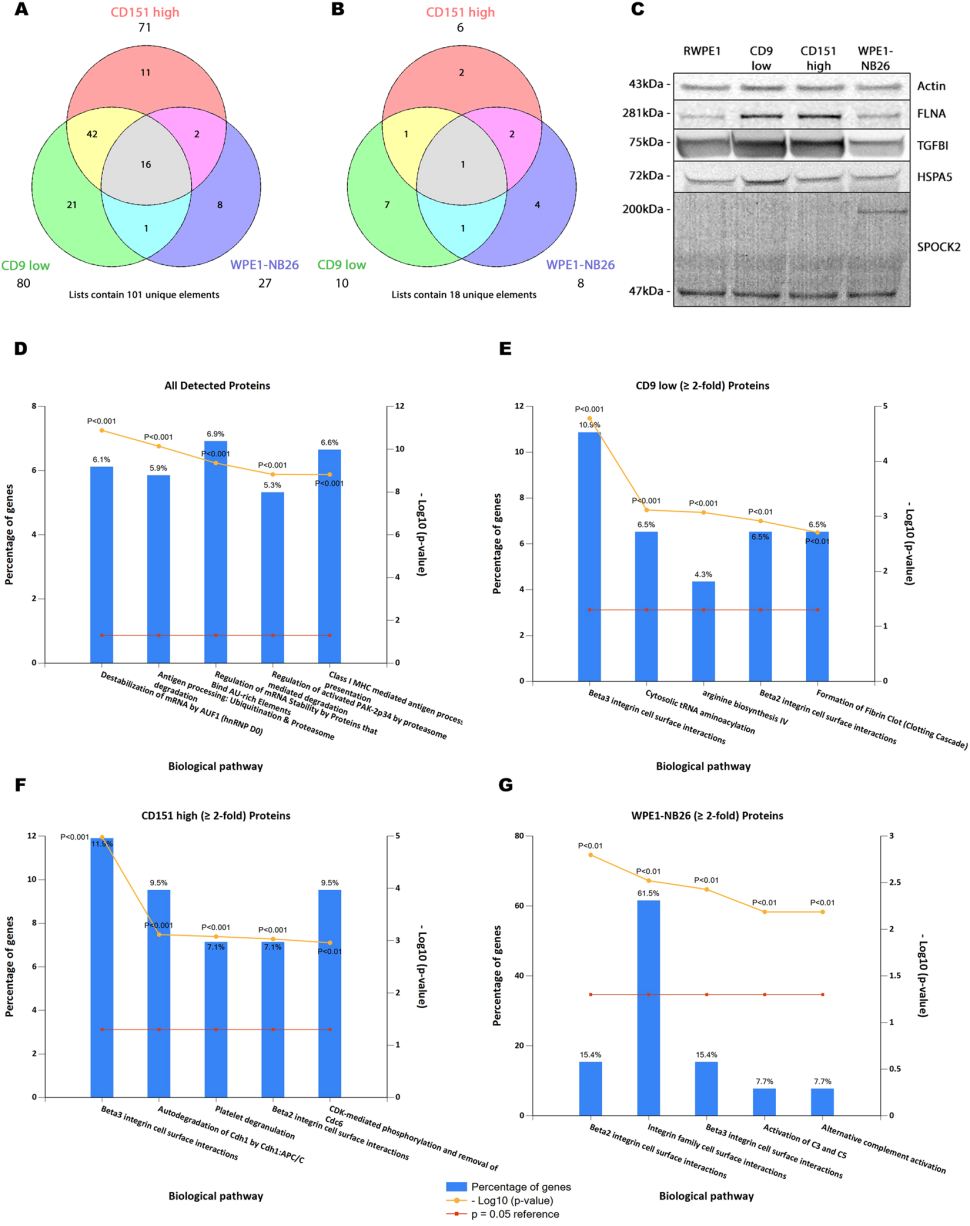
FunRich analysis of iTRAQ data. (**A**) Proteins with a ≥ 2-fold increase in expression and (**B**) a ≥ 2-fold decrease in expression when compared to RWPE1 EVs. Venn diagrams were created using the Public Research Centre for Health’s Venn diagram generator tool (http://www.bioinformatics.lu/venn.php). (**C**) Western blot analysis of Actin, Filamin-A, TGFBI, HSPA5 and SPOCK2 confirmed iTRAQ quantitated values. (**C**) Enriched biological pathways for all detected EV proteins. Degradation pathways were the predominant feature in the total dataset. (**D**) Enriched biological pathways for CD9 low EV proteins with a ≥ 2-fold increase in expression to RWPE1 EVs. (**E**) Enriched biological pathways for CD151 high EV proteins with a ≥ 2-fold increase in expression to RWPE1 EVs. (**F**) Enriched biological pathways for WPE1-NB26 EV proteins with a ≥ 2-fold increase in expression to RWPE1 EVs. Graphs were created using FunRich v2.1.2. which converts protein IDs to gene names. Western blot images are cropped, with full-length blots presented in Supplementary Fig. S8.

Since the abundance of CD9 and CD151 on EVs varied with cellular abundance, and tetraspanins have been proposed to influence EV cargo recruitment, we examined the proteome of the EVs using quantitative proteomics with isobaric tags (iTRAQ™ labels). Peptide analysis, after filtering for proteins of human origin only, detected 627 proteins that had at least one isobaric tagged peptide in common with the RWPE1 EV sample (Supplementary Fig. S1), consistent with numbers identified in other studies^27^. To determine whether the calculated protein abundance in each sample was reliable, the abundance of several traditionally used loading control proteins, including beta actin (ACTB), alpha tubulin (TBB5) and beta tubulin (TBA1B), were assessed and relatively equal expression across all samples ± 0.006 was found (Table 1). To further validate the iTRAQ peptide analysis, several proteins displaying differential expression across all samples were selected for western blot analysis (Fig. 3C). EVs from the RWPE1, CD9 low, CD151 high and WPE1-NB26 cell lines were probed for the 78 kDa glucose regulated protein (HSPA5), filamin-A (FLNA), transforming growth factor-beta induced protein ig-h3 (TGFBI) and testican-2 (SPOCK2), with total actin being used as a loading control. iTRAQ and quantitated western blot data showed similar patterns of expression, further confirming the validity of the quantitated iTRAQ values (Table 2).

**Table 1:** Traditional loading control proteins identified using iTRAQ analysis compared to RWPE1 expression.

Loading Control	RWPE1	CD9 low	CD151 high	WPE1-NB26
**Beta Actin** (ACTB)	1.000	1.055	0.858	0.915
**Beta Tubulin** (TBB5)	1.000	0.912	0.992	1.030
**Alpha Tubulin** (TBA1B)	1.000	1.026	1.132	1.043
**Average**	**1.000**	**0.998**	**0.994**	**0.996**

**Table 2:** Comparison of iTRAQ and western blot quantitated values.

Protein	RWPE1*		CD9 low		CD151 high		WPE1-NB26	
	iTRAQ	WB	iTRAQ	WB	iTRAQ	WB	iTRAQ	WB
Filamin-A (FLNA)	1.000	1.000	1.662	2.092	1.684	2.916	1.392	0.724
Transforming growth factor-beta-induced protein ig-h3 (TGFBI)	1.000	1.000	2.432	1.471	2.216	1.331	0.737	0.635
78 kDa glucose-regulated protein (HSPA5)	1.000	1.000	1.019	1.052	1.027	1.021	1.015	1.180
Testican-2 (SPOCK2)	1.000	1.000	1.209	1.066	0.913	0.966	2.564	1.455

WB = western blot.

*All values were normalised to RWPE1.

Identification of interaction networks for all detected protein IDs using FunRich revealed distinct clusters around 14-3-3 protein gamma (YWHAG) and nucleoside diphosphate kinase B (NME2) (Supplementary Fig. S2). YWHAG is thought to contribute to cancer progression and increase metastatic spread by promoting cancer cell invasion^28^. Expression ratios of YWHAG in CD9 low and CD151 high EVs indicated a selective sorting when compared to RWPE1 EVs (RWPE1:CD9 low = 1.796; RWPE1:CD151 high = 1.336), however this was not observed for WPE1-NB26 EVs (RWPE1:WPE1-NB26 = 0.800). This selective incorporation of pro-metastatic messages with cancer relevant modification of CD9 and CD151 abundance suggests that this could be involved in the promotion of early stage metastasis through the uptake of these EVs into recipient cells.

Similarly, iTRAQ expression ratios showed a decreased expression of NME2, a transcription regulator of Myc with metastasis suppressor functions^29^, across CD9 low, CD151 high and WPE1-NB26 EV populations compared to RWPE1 (RWPE1:CD9 low = 0.358; RWPE1:CD151 high = 0.620; and RWPE1:WPE1-NB26 = 0.577). This suggests that the expression of CD9 may be a contributing factor for NME2 incorporation into EVs, which could have similar effects on the progression of cancer. Further, the identification of a number of proteins involved in the epithelial-mesenchymal transition (EMT) displayed an increase in abundance in CD9 low, CD151 high and WPE1-NB26 EVs compared to RWPE1 EVs, including vimentin^30^ (RWPE1:CD9 low = 2.818; RWPE1:CD151 high = 2.265; and RWPE1:WPE1-NB26 = 1.907), moesin^31^ (RWPE1:CD9 low = 1.811; RWPE1:CD151 high = 2.022; and RWPE1:WPE1-NB26 = 1.345), and annexin A2^32^ (RWPE1:CD9 low = 1.204; RWPE1:CD151 high = 1.431; and RWPE1:WPE1-NB26 = 1.362). Taken together, these protein clusters and EMT proteins represent an enrichment for pro-tumorigenesis pathways through the negative regulation of apoptosis, Myc-related metastatic functions, and the possible promotion of EMT by EVs.

To identify proteins with large differences in abundance, the list was filtered for proteins with a ≥ 2-fold increase or decrease when compared to RWPE1 EVs. Interestingly there were far more proteins identified with increased rather than decreased abundances in any of the comparisons when compared to RWPE1. The greatest number of differences were observed for the CD9 low EVs, with 91 proteins increased and 18 decreased when compared to RWPE1. In CD151 high EVs, 77 proteins were increased and 6 decreased, and in WPE1-NB26 EVs, 35 proteins were increased and 8 decreased when compared to RWPE1 (Supplementary Tables S3 and S4). Further, visualisation of these proteins with differing abundance using Venn diagrams showed that CD9 low EVs had 28 unique proteins (21 increased and 7 decreased), CD151 high EVs had 11 unique increased and 2 unique decreased proteins, and WPE1-NB26 EVs had 8 unique increased and 4 unique decreased proteins. There were 16 increased proteins common between all three comparisons, with CD9 low and CD151 high EVs sharing a further 42 increased proteins, CD9 low and WPE1-NB26 EVs sharing 1 increased protein, and CD151 high and WPE1-NB26 EVs sharing 2 increased proteins in common (Fig. 3A). There was only 1 common protein decreased in all samples, fumarylacetoacetate hydrolase domain-containing protein 2B (FAHD2B). Due to sequence homology, FAHD2B is proposed to have hydrolase and metal binding activity, however, to our knowledge, no further characterisation has been performed on this protein. Further, CD9 low EVs share 1 protein in common each with CD151 high and WPE1-NB26 EVs, and CD151 high and WPE1-NB26 EVs share a further 2 decreased proteins (Fig. 3B).

FunRich analysis was utilized to determine enriched pathways from the iTRAQ datasets that could identify potential functions that EVs may transfer to their target cells via their protein content. Protein IDs (UniProt Accession) were imported into FunRich where they are converted into gene names for subsequent analysis. For all detected protein IDs across all samples, the biological pathways that were most highly enriched were those involved with nucleic acid destabilisation and protein degradation (Fig. 3D). These biological processes were further analysed for enrichment in the CD9 low, CD151 high and WPE1-NB26 lists with a ≥ 2-fold increase in expression compared to RWPE1. For CD9 low EVs, beta2 and beta3 integrin interaction pathways, as well as metabolic pathways were the most prevalent (Fig. 3E). Beta2 and beta3 integrin interaction pathways were enriched in CD151 high EVs, and an overall enrichment for protein degradation pathways was identified in these EVs (Fig. 3F). Integrin interaction and complement activation pathways were the most enriched pathways in WPE1-NB26 EVs (Fig. 3G). Due to the interactions that tetraspanins have with integrins, the enrichment for integrin interaction pathways was not unexpected.

### EVs display protease activity

EV involvement in the metastatic process involves crosstalk between cancer cell EVs and surrounding cells of the tumour mass. A crucial step in this process involves cellular migration and invasion through the extracellular matrix. Through iTRAQ and subsequent western blot (Fig. 3C), transforming growth factor beta-induced protein (TGFBI) was found to have a significantly increased expression in CD9 low and CD151 high EV populations. It has previously been reported that TGFBI is an integrin binding partner, capable of modulating the attachment of cells to fibronectin and collagen components of the extracellular matrix^33^. An increased expression of TGFBI in melanoma cells impaired cellular adhesion to fibronectin, collagen I and laminin, conferring strong anti-adhesive function to metastasizing cells^34^. Another function of cancer cell EVs is in the formation of the premetastatic niche; a distant site within the body that has become transformed into a more favourable environment for metastasizing tumour cells to settle and grow. This process involves the degradation of extracellular matrix components and the recruitment of various growth factors to promote cellular proliferation and neovascularization^7^. We observed a selective enrichment for the beta subunits 4, 5, 6 and 7 of the proteasome complex (PSMB4, PSMB5, PSMB6 and PSMB7 respectively) in CD9 low, CD151 high and WPE1-NB26 samples compared to RWPE1 EVs. There was also an enrichment for the proteasome activator complex subunit 2 (PSME2) in all samples, lending further evidence to the enrichment of protein degradation pathways identified by FunRich analysis (Table 3). This increase in abundance of proteins in proteasome degradation pathways in modified and tumourigenic EVs points towards possible involvement in the metastatic process.

**Table 3:** Abundance changes of proteins involved in proteasome degradation pathways.

Protein	RWPE1	CD9 low	CD151 high	WPE1-NB26
Proteasome activator complex subunit 2 (PSME2)	1.000	1.505	2.527	1.525
Proteasome subunit beta type-4 (PSMB4)	1.000	1.166	1.770	1.145
Proteasome subunit beta type-5 (PSMB5)	1.000	5.107	3.069	1.368
Proteasome subunit beta type-6 (PSMB6)	1.000	8.963	2.136	1.433
Proteasome subunit beta type-7 (PSMB7)	1.000	4.837	2.768	1.435

**Figure 4:**
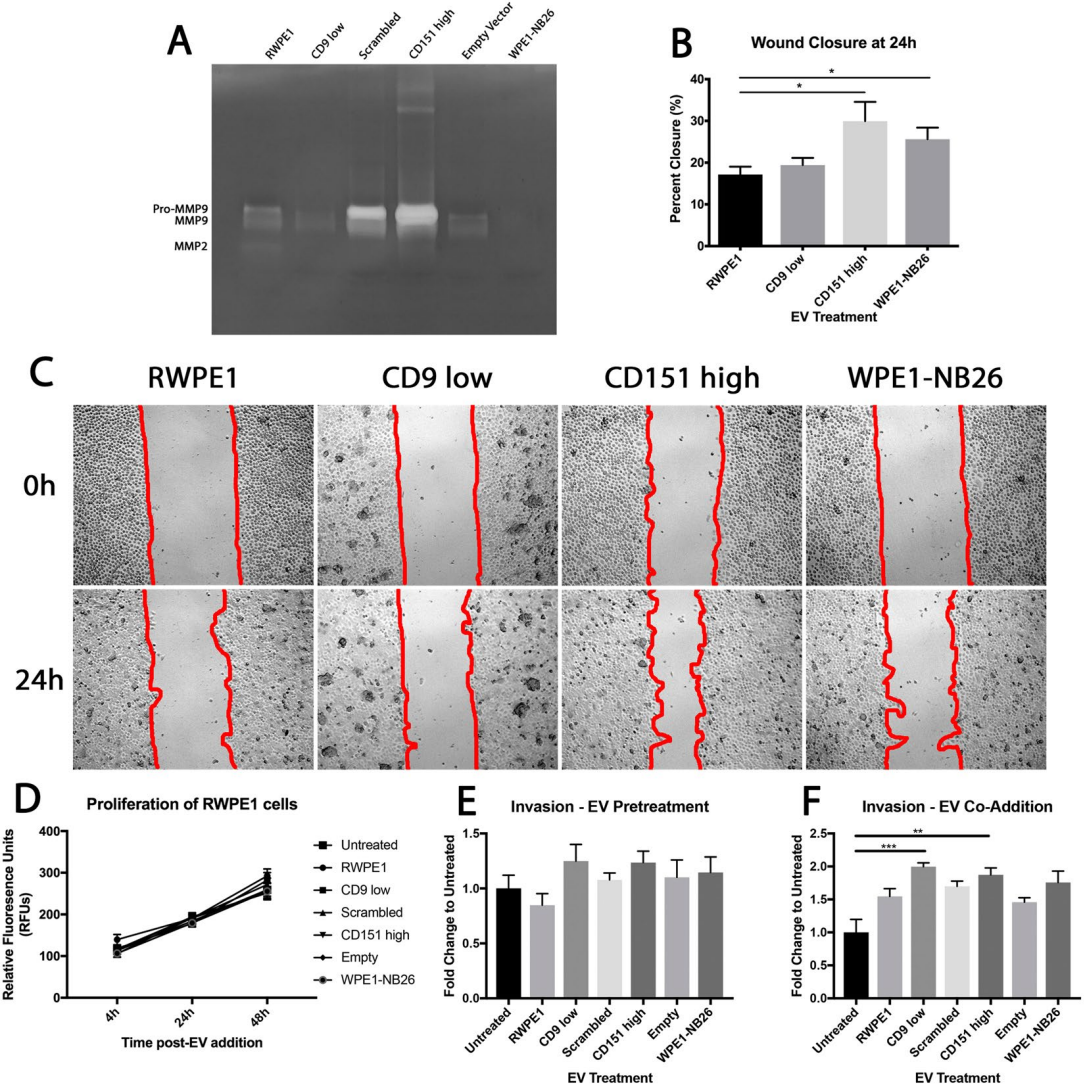
Migration and invasion of EV treated RWPE1 cells. (**A**) Gelatin zymography of EVs detected MMP2 and the pro- and active form of MMP9, with an increase in pro-MMP9 observed in Scrambled and CD151 high EVs. (**B**) Both CD151 high and WPE1-NB26 EV treated RWPE1 cells displayed enhanced wound closure using a Kruskal-Wallis test. (**C**) A wound healing assay was performed to determine the migratory capacity of RWPE1 cells treated with EVs. Red lines indicate the edge of the wound used for calculations. (**D**) A proliferation assay was performed to ensure that effects seen were not due to proliferation. No differences were seen in proliferation in EV treated RWPE1 cells. (**E**) Invasion assay of RWPE1 cells through a BME coating that was pretreated with 10 µg EVs for 24 h. No significant differences were observed compared to untreated. (**F**) Invasion assay of RWPE1 cells through a BME coating with co-addition of cells and EVs. CD9 low and CD151 high EVs significantly enhanced RWPE1 cell invasion when compared to untreated cells. Significance was determined using a Kruskal-Wallis test. All data are representative of three independent experiments. *P < 0.05; **P < 0.01; ***P < 0.001. Error bars are shown as SEM.

Matrix metalloproteinases (MMPs) are a large family of zinc-dependent endopeptidases with specificity to particular ECM substrates^35,36^. Both MMP2 (gelatinase A) and MMP9 (gelatinase B) are implicated in the progression and metastasis of many tumours via degradation of the ECM and resultant release of ECM-bound bioactive molecules that assist in angiogenesis and cellular migration^36,37^. To assess whether EVs contained active MMPs which may aid in the degradation of ECM components, gelatin and casein zymography were used. Gelatin zymography showed that all EVs possessed both pro- and active MMP9 activity, with Scrambled and CD151 high EVs displaying the highest activity, particularly for pro-MMP9. A lesser amount of active MMP2 was also detected in RWPE1 and CD151 high EV samples (Fig. 4A). Although a gelatinase, MMP9 can be detected using casein zymography if highly abundant^35^. We were able to further show increased MMP9 activity in CD151 high EVs using casein zymography (Supplementary Fig. S5). Taken together, the enrichment of various proteasome complex subunits and the expression of active MMPs, particularly in CD151 high EVs, lends evidence to support that alteration of tetraspanins in prostate cancer cells may give rise to EVs with enhanced matrix degradation properties.

### Altered CD9 and CD151 abundance on EVs affects cellular migration and invasion

EVs are able to affect target cell function via a number of mechanisms, including internalisation into the target cell via endocytosis, direct fusion with the plasma membrane to release their cargo into the cytoplasm, or by simply binding to cell surface receptors to activate internal signalling pathways^38^. CD151 has reported interactions with various integrins, including α3 and α6, which are important for CD151-related roles in many cellular processes including, in prostate cancer, migration and invasion^39^. Further, proteases, particularly MMP2 and MMP9, displayed enhanced activity in CD151 high EVs, compared to RWPE1 EVs. MMPs are known to interact with integrins and have been shown to enhance tumour cell migration and invasion through the ECM^40,41^. As integrin interactions and proteasome pathways were consistently identified using iTRAQ, we investigated whether our isolated EVs could enhance the migratory and invasive capacity of RWPE1 cells when used as a treatment.

Investigating the effects of EVs on their target cells migratory capacity using a wound-healing assay showed differential effects, dependent on the tetraspanin level. Treating RWPE1 cells with CD151 high and WPE1-NB26 EVs was able to significantly increase RWPE1 cell migration at 24 h post-EV addition when compared to RWPE1 EV-treated cells (74.88%, p = 0.0163 and 49.56%, p = 0.0331 respectively) (Fig. 4B,C). No significant differences in migration were observed by the addition of CD9 low EVs on RWPE1 cells (p = 0.9262) (Fig. 4B). To control for any effects of the EVs on cellular proliferation influencing the outcome of these assays, a proliferation assay was performed across the range of time points used for migration and invasion assays, up to 48 h. There were no significant differences in proliferation observed with any EV treatment at any time point assessed (Fig. 4D). The WPE1-NB26 cell line displays an endogenous increase in CD151 and decrease in CD9, whereas the CD151 high cell line displays an artificial increase in CD151 and decrease in CD9, with EVs from both cell lines able to provide a gain of migratory function to RWPE1 cells. However, the CD9 low cell line displays an artificial decrease in CD9 whilst retaining normal levels of CD151, with EVs from this cell line being unable to elicit a gain in migratory function to RWPE1 cells, suggesting that this gain of function is possibly due to the influence of CD151. This may be related to whether the CD151 is bound to integrin partners or whether it remains unbound in the membrane, as has been shown by Palmer *et al*. (2013), where unbound CD151 negatively influenced cellular motility when clustered on the cell surface due to increased cell-cell adhesion^42^. However, when unbound CD151 is not clustered, it was shown to enhance cellular motility^42^. Whether the CD151 on EVs is bound or unbound to integrins was not determined in this case, however, with the incredibly high levels of CD151 present in the CD151 high EVs, it would seem unlikely that it was all bound to integrins. Further, utilising super-resolution microscopy, it has been reported that the average size for tetraspanin clusters in the cell membrane is between 35–150 nm diameter^43,44^, making it unlikely that tetraspanins form clusters on EVs. The increase in cellular motility conferred by these EVs also suggests that any unbound CD151 does not become clustered in the membrane of target cells. To investigate this further, we assessed the CD151 abundance of RWPE1 cells following EV treatment (Supplementary Fig. S6), where we observed no significant differences in CD151 content, suggesting that CD151 may in fact be facilitating the interactions of partner molecules with receptors on the cell membrane to elicit their function^38^.

The ability for EVs to affect cellular invasion through the basement membrane is another key step in the metastatic cascade that integrins have been implicated in^45^, therefore we wanted to determine what impact treating RWPE1 cells with EVs arising from cells with differential CD9 and CD151 expression had on their invasive capacity. An invasion assay was performed using EV-treated RWPE1 cells on a basement membrane extract (BME) coated well of a Boyden-chamber. Pretreatment of BME with EVs (Fig. 4E) prior to cell addition did not show any significant difference in invasion compared to pretreating with PBS (untreated). Co-addition of EVs with cells (Fig. 4F) showed significant increases in invasion for both CD9 low EV (99.40%, p = 0.0002) and CD151 high EV (87.44%, p = 0.0054) treated RWPE1 cells when compared to untreated RWPE1 cells. Taken together, these results indicate that a combination of low CD9 and high CD151 expression, as seen in the CD151 high EVs, can influence migration and invasion of non-tumourigenic prostate cells. Low CD9 EV expression alone was only able to influence the invasion of a non-tumourigenic cellular population and not migration. As tetraspanins have been proposed to influence the uptake of cargo into EVs^26^, these functional differences between CD9 low and CD151 high EVs may be attributed to a selective recruitment of cargo molecules that may act on EV target cells to affect invasion and migration. Whether the functional effects seen here are due to cellular uptake of EVs and the subsequent functional effects of their cargo or EVs binding to cell surface proteins and affecting membrane signalling platform organisation requires further investigation.

Although tetraspanins themselves are commonly used as markers of EV populations, including CD63, CD9 and CD81^24^, it remains largely unexplored what alterations in tetraspanin expression on EVs has on their function and composition. An interesting observation in this study was that decreasing CD9 expression in EVs resulted in a larger number of significantly increased and decreased changes in the proteome compared to CD151 high and the tumourigenic WPE1-NB26 EVs, however this larger number of differences did not translate to an increased functional gain for RWPE1 cells when they were incubated with CD9 low EVs in the assays assessed. The tetraspanin family are thought to be important in the formation and cargo recruitment of EVs^26^, and the data presented here give evidence to support this notion, as we observed subtle but distinct differences in the proteome and functional effects of EVs with altered tetraspanin expression. However, as the number of differences in the proteome of EVs did not necessarily indicate the number of functional differences conferred by those EVs, perhaps the nucleic acid or lipid cargo, alone or in combination with the EV proteome may be key to driving the functional effects seen here, and an assessment of differences in EV cargo may elucidate the underlying molecular mechanisms involved. We have also demonstrated that CD9 and CD151 modified, and tumourigenic (WPE1-NB26) EVs can transfer metastasis-related function to a non-tumourigenic cellular population. This may have implications for tumour-derived EVs in the spread of metastatic potential to other prostate cancer cells within the primary tumour mass, priming the metastatic potential of these cells.

## Methods

### Antibodies

Mouse-anti-CD9 [1AA2], mouse-anti-CD151 [11B1c.4] and isotype controls mouse-anti-IgG1 [IB5] and mouse-anti-IgG2A [ID4.5] were a gift from C. Prof Leonie Ashman (University of Newcastle, NSW, Australia). Commercial antibodies were purchased from the following: Rabbit-anti-GAPDH (BioVision #3777R-100; Sapphire Biosciences, Redfern, NSW, Australia), mouse-anti-CD63 (BioVision #A1502-50), mouse-anti-CD82 [TS82b] (Abcam #ab59509; Abcam Australia, Melbourne, VIC, Australia), rabbit polyclonal HSPA5 (GeneTex #GTX127934; Sapphire Biosciences), FLNA (GeneTex #GTX109931), SPOCK2 (GeneTex #GTX121068) and TGFBI (GeneTex #GTX100744). Goat anti-mouse and goat anti-rabbit HRP-conjugated secondary antibodies were from Bio-Rad (Gladesville, NSW, Australia). FITC-conjugated goat anti-mouse secondary antibody was from Southern Bio-tech (*In Vitro* Technologies, Noble Park, VIC, Australia).

### Cell Lines and Cell Culture

The RWPE1 (CRL-11609) and WPE1-NB26 (CRL-2852) cell lines were purchased from American Type Culture Collection (Cryosite, South Grandville, NSW, Australia), with authentication provided with purchase, and grown in complete Keratinocyte-Serum Free Media (K-SFM) (Gibco, Thermo Fisher Scientific, Mulgrave, VIC, Australia) at 37 °C/5% CO_2_. RWPE1 cells were transfected to display either low CD9 or high CD151 expression as detailed below.

### Generation of CD151 overexpressing and CD9 knockdown RWPE1 cell lines

For overexpression of CD151, 2.5 µg of pcDNA3.1/Zeo vector (Invitrogen, Thermo Fisher Scientific) containing the full length human CD151 sequence^39^ was transfected into RWPE1 cells with Lipofectamine LTX (Invitrogen). Cells were selected with 500 µg/mL Zeocin (Gibco) to facilitate the production of a stable CD151 overexpressing cell line, herein referred to as CD151 high. An empty pcDNA3.1/Zeo vector control line was also generated in this manner, herein referred to as Empty Vector.

Stable knockdown of CD9 in RWPE1 cells was conducted as described above using the pRS CD9 shRNA vector (Origene Cat# TR314060; Australian Biosearch, Karrinyup, WA, Australia) and selection with 1.5 µg/mL Puromycin (Sigma Aldrich, Castle Hill, NSW, Australia) to achieve stable knockdown of CD9, herein referred to as CD9 low. The supplied scrambled shRNA vector (Origene) was also used to generate a control cell line, herein referred to as Scrambled.

### Extracellular Vesicle Isolation

EVs were isolated from the media of cells following a 72 h incubation using a modified ultrafiltration procedure as previously described^46^. Briefly, 100 mL of spent media per collection was sequentially passed through 0.22 µm PES and 0.1 µm PVDF syringe filters (Merck Millipore, Kilsyth, VIC, Australia). Clarified media was then placed in a 100 kDa centrifugal filter unit (Merck Millipore) and centrifuged at 4,000 × *g*, 4 °C until sample had passed through. The flowthrough was discarded and the retentate washed with 0.1 µm filtered sterile PBS twice to yield a retentate of concentrated EVs in PBS. Retentates were collected and stored at −30 °C until required.

### Nanoparticle Tracking Analysis

Nanoparticle tracking analysis (NTA) was used to determine particle size distribution and concentration of EV samples from RWPE1, CD9 low, CD151 high and WPE1-NB26 cell lines. Concentrated EV suspensions were diluted 1:1000 in PBS before introduction into the sample chamber of a Nanosight NS300 NTA system (Malvern, ATA Scientific, Taren Point, NSW, Australia) using a syringe pump (Harvard Apparatus, ATA Scientific) on a speed setting of 50. The NS300 was adjusted so that particles were in the focal plane and a scientific CMOS camera captured 3 × 60 s videos per sample. Post-acquisition analysis settings were kept constant between samples. Particle size distribution and concentration were determined using NTA v2.3 software (Malvern).

### Flow Cytometry

Cell surface CD9 and CD151 protein levels of cell lines were determined using a modified flow cytometry procedure as previously described^47^. Briefly, cells were washed and resuspended in NRS/PBA (10% normal rabbit serum (Invitrogen), 0.1% BSA, 0.1% sodium azide in PBS) containing either CD9, CD151 or combined IgG1/IgG2A primary antibodies at 6 µg/mL. Cells were washed in PBA and resuspended in NRS/PBA containing FITC-conjugated secondary antibody. Cells were washed and fixed prior to analysis using a BD FACScalibur flow cytometer using Cellquest Pro^®^ software (both BD Biosciences, North Ryde, NSW, Australia). Expression of CD9 and CD151 was analysed using Weasel software (WEHI, Melbourne, VIC, Australia) and normalized to their respective isotype controls.

### Western Blot

Lysis, protein quantitation and western blotting of cells and EVs were performed as previously described^47^, with some modifications. Briefly, cells were lysed in a 1% NP-40 lysis buffer with cOmplete Protease Inhibitors (Roche, Sigma Aldrich) and sonicated 2 × 10 s on ice. Protein concentrations were determined using a micro BCA assay (Thermo Scientific) according to the manufacturer’s instructions. Cell and EV lysates were prepared in a reducing or non-reducing (without 2-mercaptoethanol) sample buffer (50 mM Tris-HCl pH 6.8, 5% glycerol, 2% SDS, 1.5% 2-mercaptoethanol with bromophenol blue) and heated at 70 °C, 5 min. Lysates (10 µg) were electrophoresed and transferred to nitrocellulose, followed by incubation in Ponceau-S.

Non-reduced blots were probed with primary antibodies CD9 (4 µg/mL), CD151 (2 µg/mL) and CD82 (1:2000), with cell blots being further probed for GAPDH (1:1000) for quantitation of tetraspanin expression, and EV blots being further probed for CD63 (1:1000) expression. Reduced blots were probed with ACTB (1:10000), TGFBI (1:1000), FLNA (1:1000), HSPA5 (1:1000) and SPOCK2 (1:500). After secondary incubation, blots were developed using an ECL detection reagent. Blots were scanned using an Image Quant LAS-4000 Smart Lightbox or an Amersham Imager 600 (GE Healthcare Australia, Parramatta, NSW, Australia). Bands were quantitated using ImageJ v1.47 software (http://imagej.nih.gov).

### Peptide Preparation and iTRAQ Labelling

Isolated RWPE1, CD9 low, CD151 high and WPE1-NB26 EVs pooled from three separate replicate EV preparations, equivalent to 150 µg, were suspended in ice-cold 0.1 M Na_2_CO_3_, pH 11, with cOmplete Protease and PhosSTOP phosphatase inhibitors (Roche), sonicated and incubated at −20 °C overnight. Samples were concentrated using a 10 kDa centrifugal filter unit (Merck Millipore) and washed with 50 mM triethylammonium bicarbonate (TEAB). Samples were dissolved in urea mix (6 M urea, 2 M thiourea) and reduced with 20 mM dithiothreitol (DTT) for 45 min at 37 °C followed by alkylation with 40 mM iodoacetamide for 1 h at room temperature. Proteins were first digested with 0.04 AU of Lysyl Endopeptidase C (Wako, Japan) for 3 h at room temperature followed by digestion with 5 µg of trypsin (after dilution of the urea mix to below 1 M urea), 0.3 M thiourea with TEAB, and incubated at 37 °C overnight^48^. Peptides were spun through the 10 kDa filter unit and acidified with formic acid to precipitate lipids. Lipids were removed by centrifugation at 20,000 × g for 20 min. Peptides were desalted using in-house made reverse phase microcolumns as previously described^49^. Briefly, microcolumns consisted of a modified gel loading pipette tip with a C18 Empore Disk plug (Sigma Aldrich) packed with a 1 cm layer of Poros Oligo R3 reverse phase resin (Applied Biosystems; Thermo Fisher Scientific) dissolved in 70% acetonitrile. Columns were equilibrated using 0.1% trifluoracetic acid (TFA) and samples slowly passed through the resin. Columns were washed with 0.1% TFA and bound peptides eluted using 50% acetonitrile/0.1% TFA followed by 70% acetonitrile/0.1% TFA.

Purified EV peptide samples were quantitated using a Qubit 2.0 Fluorometer (Thermo Fisher Scientific) and 50 µg of peptide from each sample used for iTRAQ^TM^ labelling. iTRAQ^TM^ 4-plex labels (Applied Biosystems) were added to lyophilised peptide samples as per the manufacturer’s instructions. Labelled peptides were quantitated and mixed in a 1:1:1:1 ratio determined using a micrOTOF-Q II (Bruker Biosciences, Preston, VIC, Australia) coupled to a Dionex Ultimate 3000RSLC nanoflow HPLC system (Thermo Fisher Scientific).

### Tandem Mass Spectrometry

iTRAQ™ labelled peptides from extracellular vesicles were separated offline using hydrophobic interactions chromatography (HILIC)^50^. Peptides were dried after HILIC separation and resuspended in 5 µL of 0.1% TFA. Peptides were loaded on an Acclaim PepMap100 C18 Nano-trap column (2 cm × 100 µm, 5 µm, 100 Å) using 0.1% TFA and separated on an in-house packed Reprosil-Pur C18-AQ (3 µm; Dr. Maisch GmbH) analytical column (20 cm × 75 µm) using a Dionex Ultimate^®^ 3000 Nano LC system (Thermo Scientific) and eluted at a flow of 250 nL/min. Peptides were analysed an Orbitrap Fusion (Thermo Fisher Scientific). Survey scans of peptide precursors from 350 to 1400 *m*/*z* were performed at 120,000 resolution (at 200 *m*/*z*) with a 5 × 10^5^ ion count target, the max injection time was 60 ms. HCD fragmentation with a normalized collision energy of 38, and MS2 analysis in the Orbitrap. The MS2 resolution was set to 30,000 and the max injection time was 100 ms. Only those precursors with charge state +2, +3, and +4 were sampled for MS2^51^.

### Bioinformatic Analysis

Raw LC-MS/MS data files were analysed using Proteome Discoverer (Version 1.4.1.14, Thermo Fisher Scientific), employing database searches using both an in-house Mascot server (Version 2.2.04, Matrix Science, London, UK), and the embedded Sequest HT server with the following criteria: database, SwissProt Homo Sapiens protein database (updated 15-01-2015, 20193 sequences), with common contaminants; enzyme, trypsin; maximum mussed cleavages, 2; variable modifications included oxidation of Met (M), iTRAQ 4plex, acetyl (protein N terminus) and Lys (K), deamidation for Asn (N) and Gln (Q), and carbamidomethylation of Cys (C) residues as a static modification. The MS and MS/MS results were searched with a precursor mass tolerance of 10 ppm, and an MS/MS mass tolerance of 0.05 Da. The Mascot results were filtered in Proteome Discoverer using Percolator to ensure the false discovery rate was less than 0.01. Peptides with different amino acid sequences or modifications were considered unique. Data were further analysed using FunRich v2.1.2^52^, an open access functional enrichment and interaction network tool, to further identify key biological processes and pathways in samples.

### Zymography

Protease activity of EVs was assessed using 12% casein and 10% gelatin zymogram gels (Thermo Fisher) as per the manufacturer’s instructions. Briefly, three individually isolated EV samples for each cell line (10 µg) were prepared in a non-reducing sample buffer and run on gels in Tris-Glycine SDS running buffer (Thermo Fisher). Proteases were renatured and developed before staining with Colloidal Blue Staining Kit (Thermo Fisher). Destaining was performed as per the manufacturer’s instructions.

### Proliferation Assay

Cellular proliferation following treatment with EVs was assessed using a Resazurin assay as previously described^47^. Briefly, RWPE1 cells were seeded into a 96-well plate. After an overnight incubation, 10 µg/mL of RWPE1, CD9 low, Scrambled, CD151 high, Empty Vector and WPE1-NB26 EVs were added to wells in growth factor-free K-SFM. A resazurin mix (final concentration 300 µM resazurin, 40 µM methylene blue, 1 mM potassium hexacyanoferrate (III), 2 mM potassium hexacyanoferrate (II) trihydrate) was added to wells at 0, 20 and 44 h and incubated for 4 h. Proliferation at 4, 24 and 48 h was assessed using a fluorescence plate reader at 544 nm excitation with 590 nm emission wavelengths.

### Wound Healing Assay

Wound healing assays were conducted on RWPE1 cells using a modified protocol as previously described^53^. Briefly, RWPE1 cells were grown as confluent monolayers in 12-well plates and scratched using a P200 tip. Cellular debris from the wound was removed by washing twice in sterile PBS. Growth factor-free K-SFM, containing 10 µg/mL of EVs (as determined by BCA assay), was added to wells. Wounds were photographed hourly for 24 h using a Zeiss Axiovert 200 M microscope with a Zeiss Incubator XL-3 489 attachment (Carl Zeiss, North Ryde, NSW, Australia) maintaining cells at 37 °C/5% CO_2_. Cellular migration was measured using AxioVision v4.9.1 software (Carl Zeiss) by measuring the size of the wound at each time point. Three individual measurements were made on each wound and averaged. Three independent experiments with a minimum of three replicates per experiment were pooled. Wound closure was calculated as percentage wound closure from *t* = 0.

### Invasion Assay

Cellular invasion was assessed using a 96-well Cultrex Invasion Assay kit (Trevigen; Bio-Scientific, Gymea, NSW, Australia). A 250 µg/mL solution of Cultrex Reduced Growth Factor Basement Membrane Extract (Trevigen) was prepared and 50 µL added to the inserts of a Boyden chamber and incubated at 37 °C overnight. Membranes were either pretreated with EVs for 24 h before seeding cells or had EVs added at the time of cell seeding. For pretreated wells, 50 µL of EVs (10 µg/mL in growth factor-free K-SFM) were added to membranes and incubated at 37 °C for 24 h. RWPE1 cells were added to the top chambers at 5 × 10^4^ cells per well on top of the EV solution for pretreated wells or added with EVs for co-addition wells (100 µL total volume per invasion chamber well). Complete K-SFM was added to bottom chambers. Plates were incubated for 48 h to assess invasion. Wells were washed, and invasive cells dissociated from the membranes and stained with Calcein-AM using the supplied reagents as per the manufacturer’s instructions. Calcein-AM fluorescence was detected using a fluorescence plate reader at 485 nm excitation with 520 nm wavelengths.

### Statistical analysis

One-way ANOVA, with Bonferroni’s multiple comparisons test, was used for analysis of western blot flow cytometry and NTA data. Wound healing assay and invasion assay data were analysed using a Kruskal-Wallis test with Dunn’s multiple comparisons. Two-way ANOVA with Bonferroni’s multiple comparisons was used for analysis of proliferation data. Differences were considered statistically significant for P-values < 0.05. All analyses were performed using GraphPad Prism v6.0.4 software (GraphPad, San Diego, CA, USA). Data are presented as the mean ± standard error of the mean (SEM).

## Electronic supplementary material

Supplementary Information
