# Supplementary material for: Extracellular vesicles with altered tetraspanin CD9 and CD151 levels confer increased prostate cell motility and invasion
Source: Sci Rep. 2018 Jun 11;8:8822. doi: 10.1038/s41598-018-27180-z (PMC5995928; doi:10.1038/s41598-018-27180-z)
Supplement: Supplementary file 1 — Supplementary Information [file 41598_2018_27180_MOESM1_ESM.pdf]

Extracellular vesicles with altered tetraspanin CD9 and CD151 levels confer increased prostate cell motility and invasion

Joshua S. Brzozowski[1,2], Danielle R. Bond[1,3], Helen Jankowski[1,2], Belinda J. Goldie[1,4], Rachel Burchell[1,2], Crystal Naudin[2,5], Nathan D. Smith[6], Christopher J. Scarlett[1,3], Martin R. Larsen[7], Matthew D. Dun[1,2], Kathryn A. Skelding[1,2], Judith Weidenhofer[1,2]*

[1] Cancer Research Program, Hunter Medical Research Institute, New Lambton, NSW, Australia

[2] School of Biomedical Sciences and Pharmacy, The University of Newcastle, Callaghan, NSW, Australia

[3] School of Environmental and Life Sciences, The University of Newcastle, Ourimbah, NSW, Australia

[4] Department of Biochemistry and Molecular Biology, Monash Biomedicine Discovery Institute, Monash University, Clayton, VIC, Australia

[5] Emory University, Atlanta, Georgia, USA

[6] ABRF, Research Services, University of Newcastle, Callaghan, NSW, Australia

[7] Department of Biochemistry and Molecular Biology, University of Southern Denmark, Odense, Denmark

* Correspondence to Judith.Weidenhofer@newcastle.edu.au

Supplementary Figure S1: iTRAQ data was transformed using the equation Y=1/Y and abundances were plotted relative to RWPE1 using Microsoft Excel. **(A)** Comparison of RWPE1 and CD9 low EV protein abundances. **(B)** Comparison of RWPE1 and CD151 high EV protein abundances. **(C)** Comparison of RWPE1 and WPE1-NB26 EV protein abundances.

Supplementary Figure S2: Interaction network of all identified proteins. FunRich v2.2.1 interaction analysis revealed distinct clusters of proteins. 14-3-3 protein gamma (YWHAG) and nucleoside diphosphate kinase B (NME2) are highlighted. YWHAG is implicated in metastatic pathways and was enriched in tetraspanin-modified EVs compared to normal prostate EVs. NME2 has been identified as having tumour suppressor functions, and showed decreased expression in all tetraspanin-modified and WPE1-NB26 EVs compared to normal prostate EVs.

Supplementary Figure S5: Casein zymography detected MMP9 in CD151 high EVs, further highlighting the enhanced matrix degradation properties of these EVs.

Supplementary Figure S6: Determination of CD9 and CD151 content in 24h EV-treated RWPE1 cells. RWPE1 cells were treated with EVs, a PBS control, or no treatment (media only) for 24h before cells were lysed and used for western blot. CD9 and CD151 content of RWPE1 cells was assessed and no significant differences were seen in CD9 or CD151 abundance with any treatment compared to RWPE1 EV treated cells. There was a significant decrease in CD9 abundance in Empty Vector

(p=0.0308) and WPE1-NB26 (p=0.0223) EV treated cells compared to media-only cells. Full length blots are also supplied in Supplementary Figure S9.

Supplementary Figure S7: Full length blots for Figure 2.

Supplementary Figure S8: Full length blots for Figure 3.

Supplementary Figure S9: Full length blots for Supplementary Figure S6.

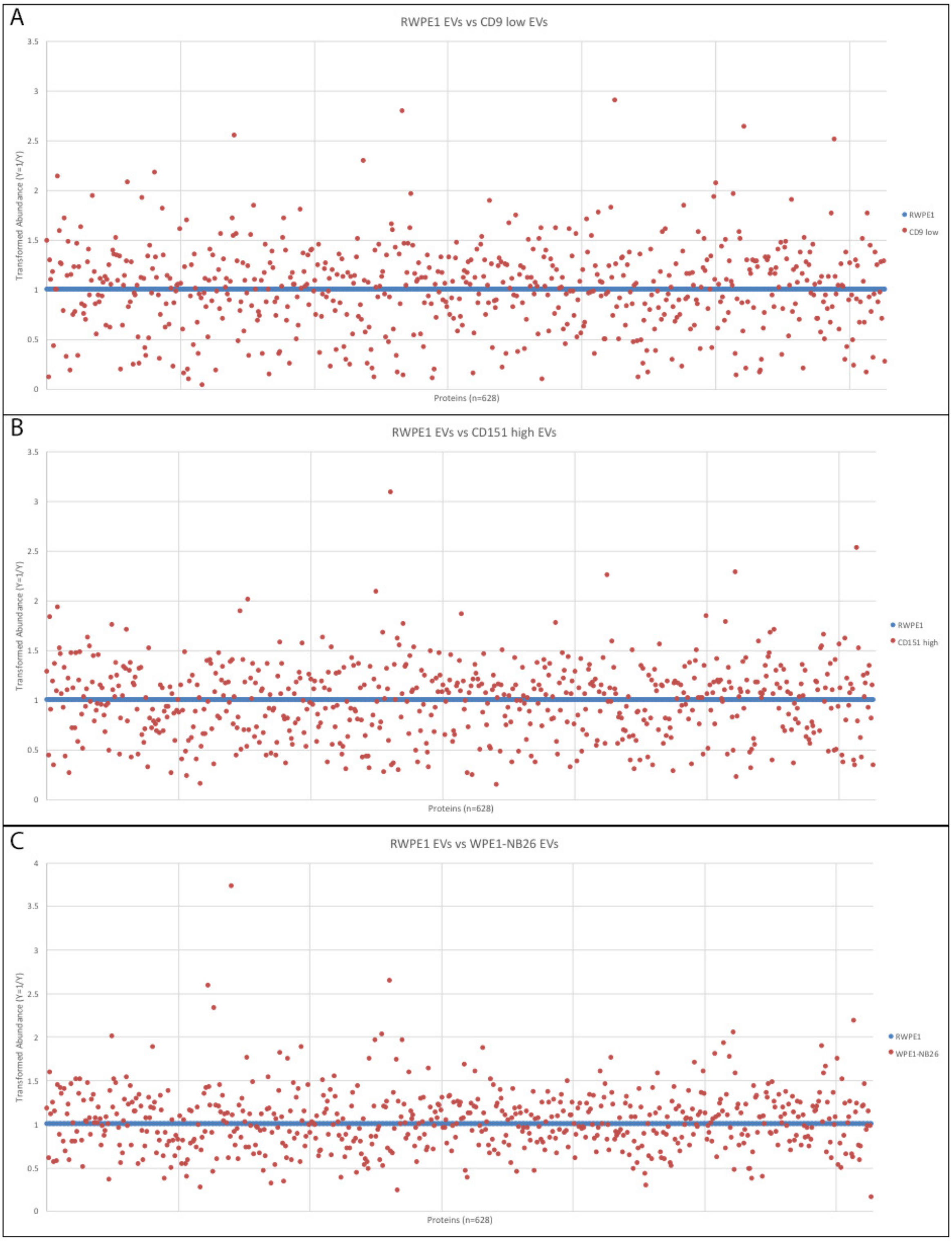

Supplementary Figure S1

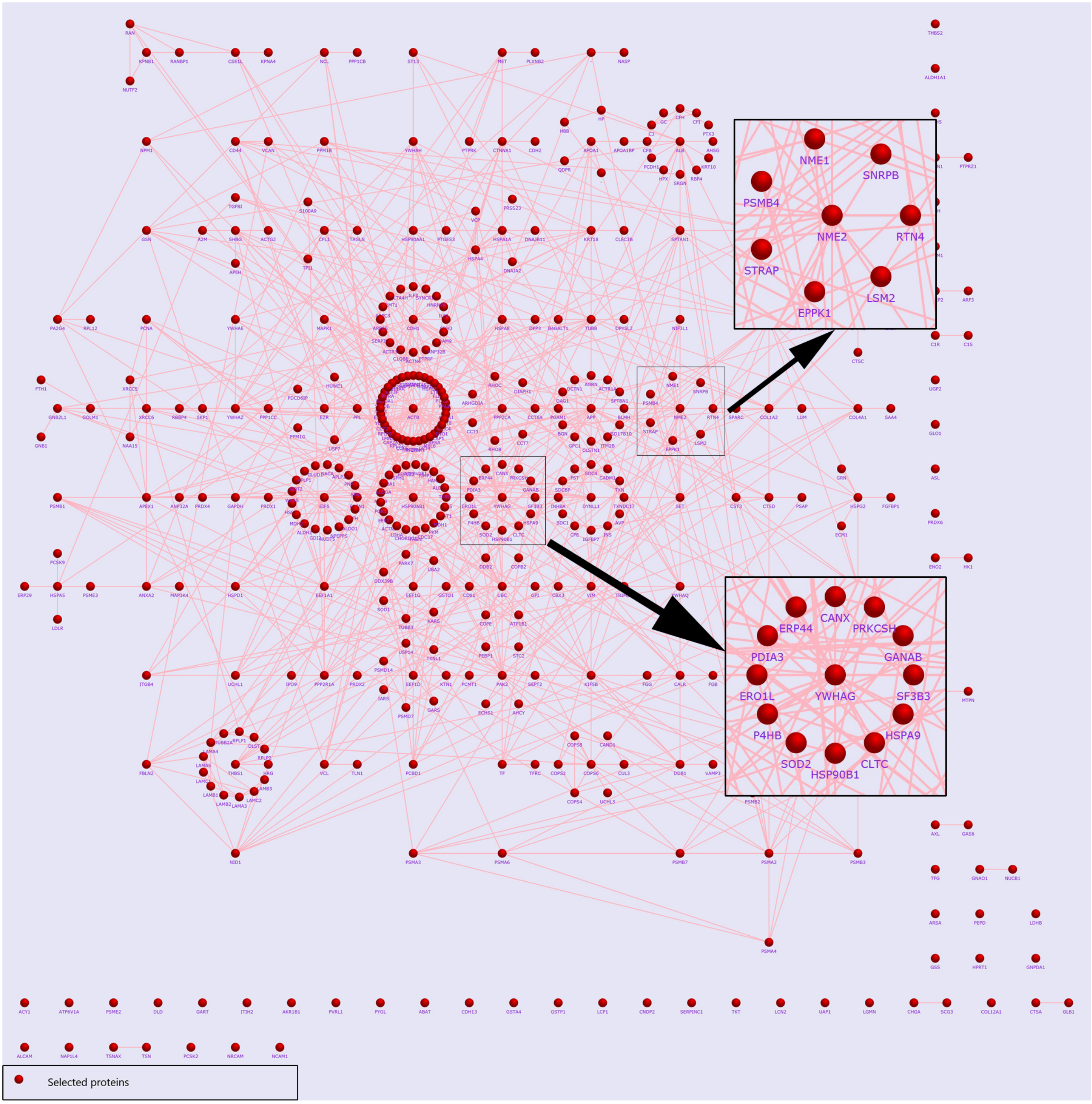

Supplementary Figure S2

Supplementary Table S3: Proteins detected by iTRAQ that displayed a ≥ 2-fold increase in expression in at least one sample compared to RWPE1.

Accession	Gene Name	CD9 low	CD151 high	WPE1-NB26
P02794	FTH1	21.613	6.239	3.714
P02679	FGG	10.379	4.246	2.560
P53365	ARFIP2	10.277	2.701	2.172
P28072	PSMB6	8.963	2.136	1.433
Q05066	SRY	8.354	2.567	1.561
A0PJA6	TF	7.994	2.248	1.660
P16870	CPE	7.988	3.130	2.049
Q52MB2	C12orf68	7.303	2.198	1.392
P23142	FBLN1	7.286	3.344	4.197
P08123	COL1A2	6.854	2.267	1.599
P02462	COL4A1	6.468	2.487	1.871
P35542	SAA4	6.251	3.684	2.166
Q9ULF5	SLC39A10	6.046	2.873	1.624
Q08257	CRYZ	6.041	2.511	1.383
P22102	GART	5.763	2.707	1.514
Q8IWU5	SULF2	5.761	2.122	1.151
E0WMV9	HLA-B	5.410	3.745	1.711
P04424	ASL	5.344	2.816	2.230
Q8N0Y7	PGAM4	5.254	3.182	2.043
P28074	PSMB5	5.107	3.069	1.368
O75106	AOC2	5.072	2.200	1.338
P02675	FGB	4.949	2.064	1.862
Q99436	PSMB7	4.837	2.768	1.435
Q6PIQ7	IGL@	4.832	4.465	2.091
P16519	PCSK2	4.694	2.314	1.768
P43490	NAMPT	4.684	6.683	1.436
P01185	AVP	4.432	3.787	2.028
P13284	IFI30	4.413	2.676	0.858
Q13838	DDX39B	4.391	3.449	1.917
Q9UBR2	CTSZ	4.133	2.645	2.036
O76061	STC2	4.030	2.341	1.334
P14550	AKR1A1	3.999	3.265	1.609
O95084	PRSS23	3.925	2.231	1.848
P16112	ACAN	3.923	2.367	1.521
Q06828	FMOD	3.881	3.318	2.064
P09488	GSTM1	3.836	2.713	1.353
Q9Y6R4	MAP3K4	3.591	2.901	6.409
Q9NQP4	PFDN4]	3.423	2.047	1.653
Q13443	ADAM9	3.352	1.779	1.662
P13987	CD59	3.344	2.213	2.609
Q8N6C5	IGSF1	3.323	1.980	2.042
P00740	F9	3.307	1.260	1.114
Q9Y2E5	MAN2B2	3.201	2.364	1.713
D6R904	TPM3	3.103	2.325	1.461
K7ERG3	TPM4	2.994	1.717	1.106
P22061	PCMT1	2.985	2.885	1.451
P00367	GLUD1	2.967	1.411	1.359
P07237	P4HB	2.945	1.873	1.374
Q92820	GGH	2.883	2.537	1.110
P08670	VIM	2.818	2.265	1.907

Supplementary Table S3 Continued:

Accession	Gene Name	CD9 low	CD151 high	WPE1-NB26
P47756	CAPZB	2.818	2.066	1.538
P63098	PPP3R1	2.818	1.535	1.186
Q4ZGM8	HBA2 (mutant)	2.813	0.561	0.519
P07951	TPM2	2.802	1.741	1.512
P02787	TF	2.800	2.455	1.369
P12081	HARS	2.765	1.734	1.198
P08758	ANXA5	2.676	1.761	0.952
P49588	AARS	2.651	2.650	1.824
Q12841	FSTL1	2.614	2.372	3.431
Q08174	PCDH1	2.604	1.137	1.819
P13611	VCAN	2.535	1.800	1.552
P16298	PPP3CB	2.531	2.305	1.682
Q15046	KARS	2.460	2.785	1.337
P50502	ST13	2.459	2.061	2.209
Q15582	TGFBI	2.432	2.216	0.737
P40925	MDH1	2.420	1.341	1.072
P00352	ALDH1A1	2.410	1.767	1.721
Q9NU22	MDN1	2.373	1.991	1.018
P26641	EEF1G	2.343	2.682	1.783
P13929	ENO3	2.335	1.788	1.152
B4DKT2	CUL4A*	2.318	2.919	1.787
P02765	AHSG	2.273	1.619	1.288
P60660	MYL6	2.224	2.200	1.499
P19883	FST	2.130	3.666	1.939
Q03154	ACY1	2.119	1.447	1.153
P06744	GPI	2.085	2.242	1.335
Q04917	YWHAH	2.062	1.589	1.118
Q9UBP4	DKK3	2.028	2.239	1.299
Q06481	APLP2	2.025	1.558	0.933
P98095	FBLN2	2.009	1.014	1.432
P35908	KRT2	1.161	3.973	0.812
P61604	HSPE1	1.911	3.065	1.380
P00441	SOD1	1.945	3.063	1.100
Q12906	ILF3	1.431	2.906	1.681
P62158	CALM1	1.595	2.569	1.649
Q9UL46	PSME2	1.505	2.527	1.525
P52758	HRSP12	1.818	2.286	1.244
P08476	INHBA	1.146	2.183	3.151
Q9HC38	GLOD4	1.664	2.086	1.480
P62805	HIST1H4A	1.035	2.053	0.811
P26038	MSN	1.811	2.022	1.345
Q6LAM1	CFI	1.725	2.019	1.180
Q43707	ACTN4	1.613	2.016	2.766
P09486	SPARC	1.213	1.241	2.930
Q8NBJ4	GOLM1	1.644	1.635	2.679
P01024	C3	1.172	1.138	2.660
P35556	FBN2	1.772	1.380	2.602
Q92563	SPOCK2	1.209	0.913	2.564
Q12805	EFEMP1	1.662	1.322	2.539
P15514	AREG	1.439	1.239	2.280
P07996	THBS1	1.142	1.496	2.145

*cDNA FLJ58013, highly similar to Cullin-4A

Supplementary Table S4: Proteins detected by iTRAQ that displayed a ≥ 2-fold decrease in expression in at least one sample compared to RWPE1.

Accession	Gene Name	CD9 low	CD151 high	WPE1-NB26
P80188	LCN2	0.345	0.444	0.685
P22392	NME2	0.358	0.620	0.577
Q6P2I3	FAHD2B	0.380	0.439	0.487
P06702	S100A9	0.392	0.715	0.268
Q9H3G5	CPVL	0.398	0.605	0.527
P15531	NME1	0.437	0.735	0.697
P00505	GOT2	0.459	1.103	0.530
B4DVL8	HEXA*	0.468	0.518	0.690
O75794	CDC123	0.481	0.588	0.650
Q16222	UAP1	0.484	0.946	0.890
P21810	BGN	0.701	0.324	0.379
Q9UNW1	MINPP1	0.566	0.395	0.457
P18827	SDC1	0.972	0.480	0.511
P07339	CTSD	0.644	0.498	0.570
P04264	KRT1	0.714	1.018	0.387
P04792	HSPB1	1.016	0.927	0.430
P19835	CEL	0.746	0.595	0.492
O60502	MGEA5	0.718	0.569	0.498

* cDNA FLJ50884, highly similar to Beta-hexosaminidase alpha chain (EC 3.2.1.52)

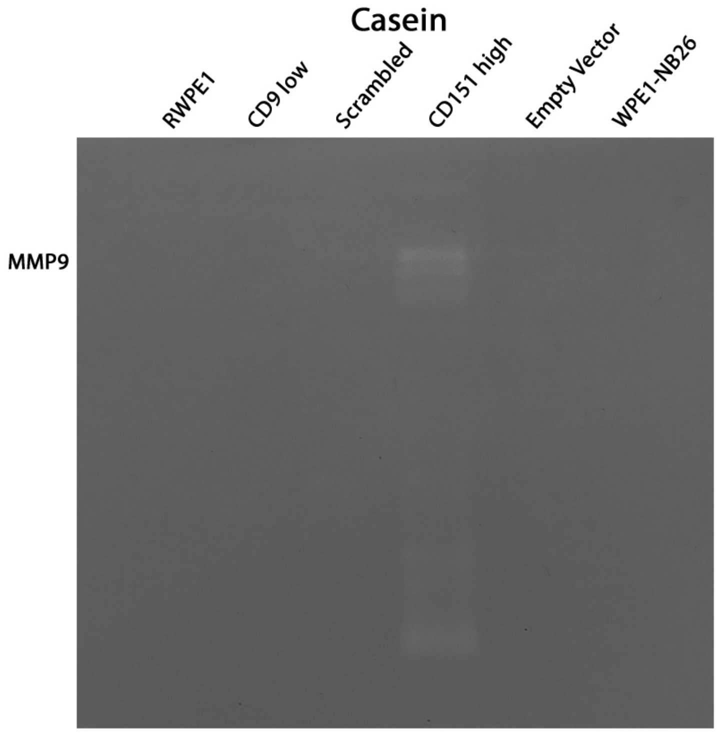

Supplementary Figure S5

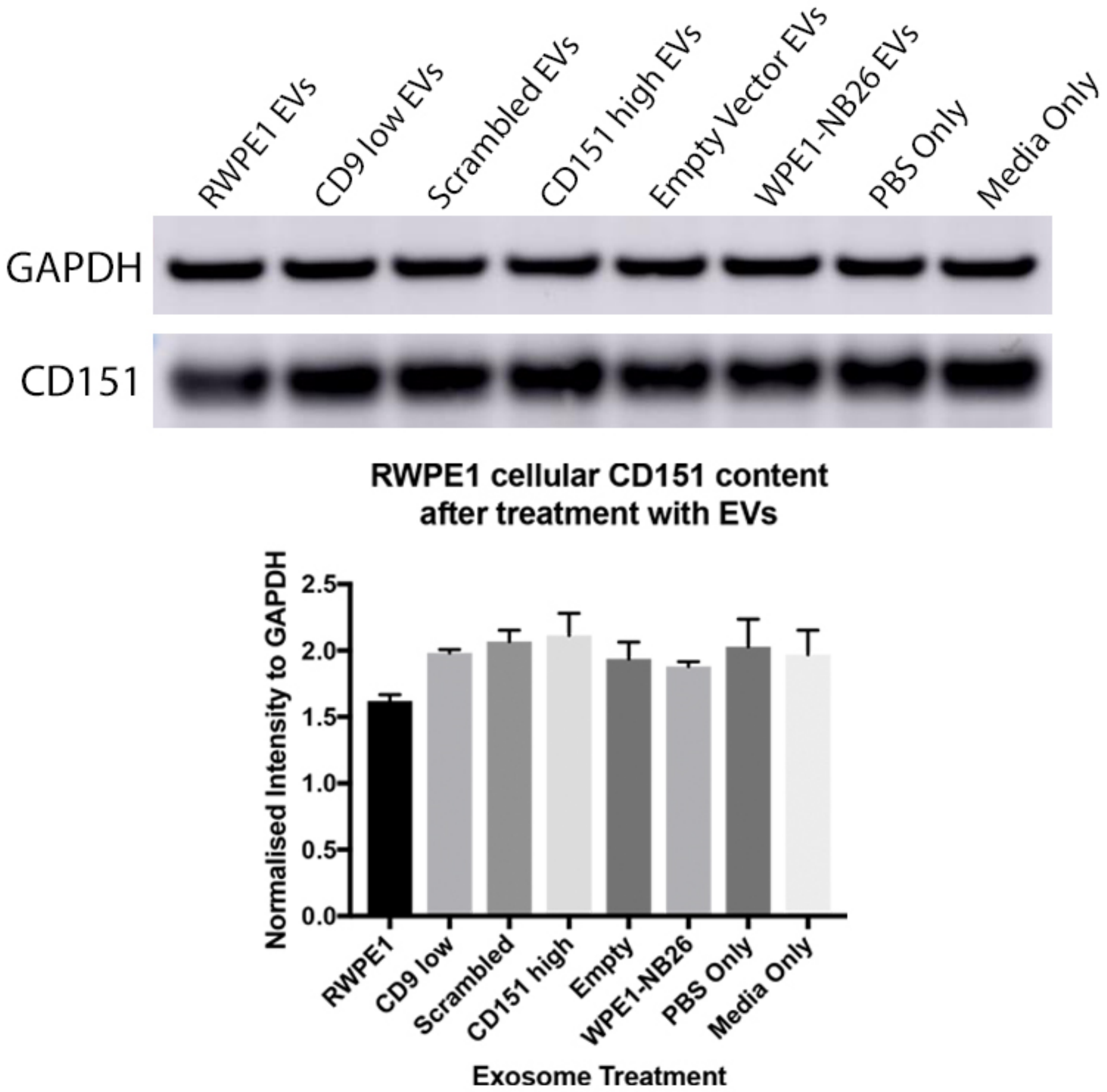

Supplementary Figure S6

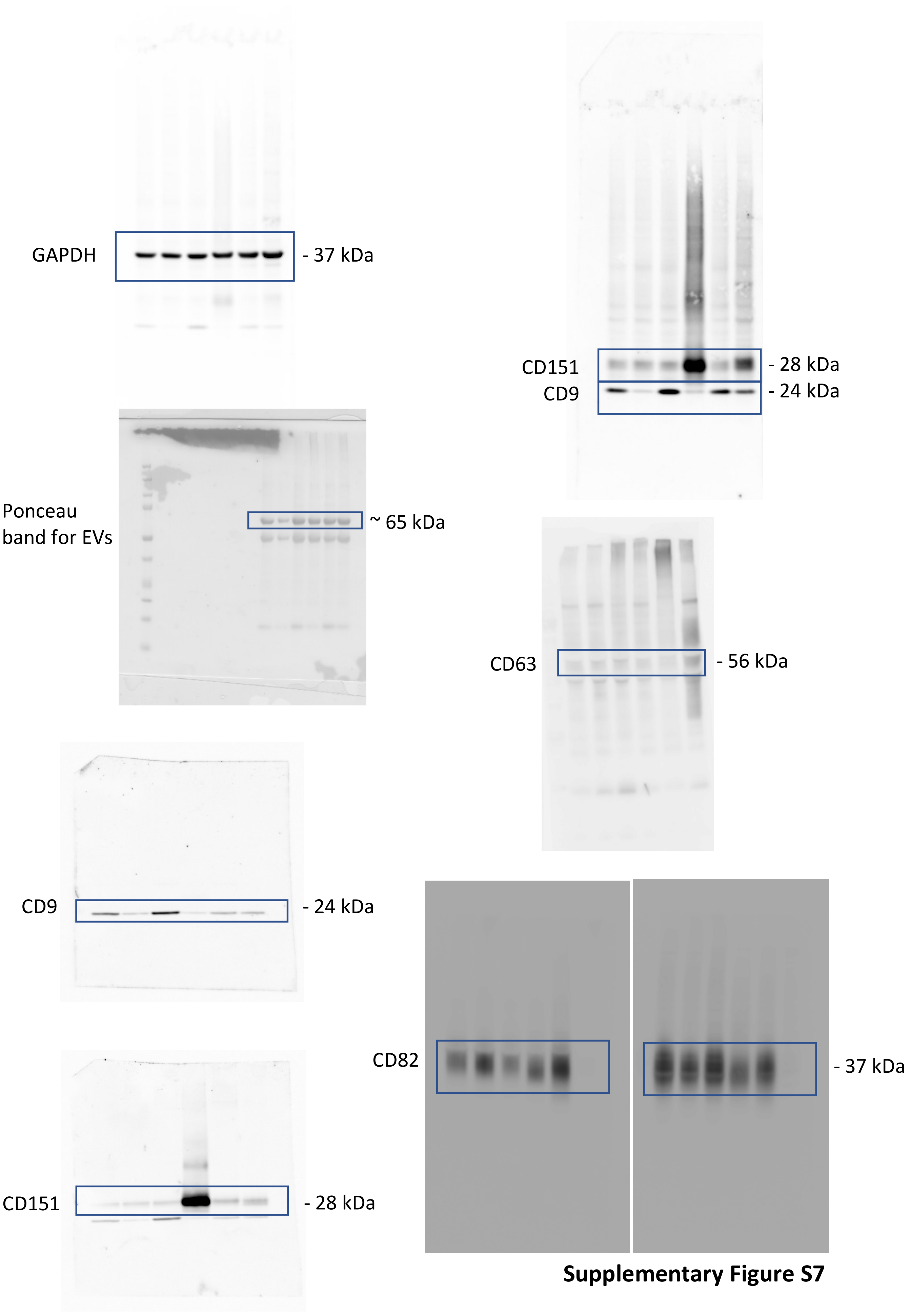

Supplementary Figure S7

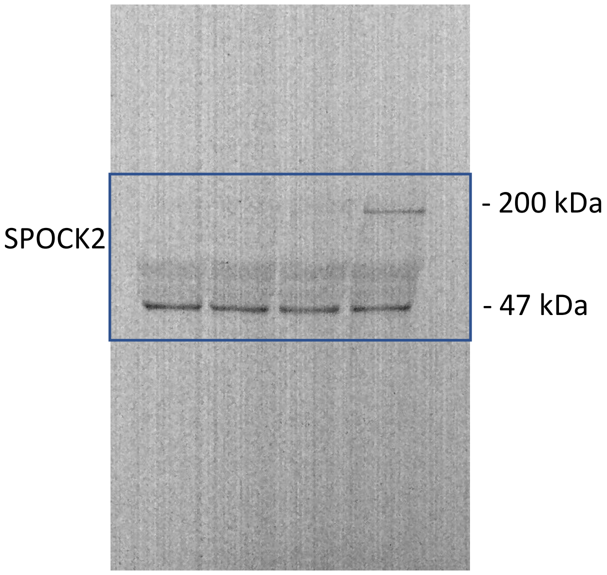

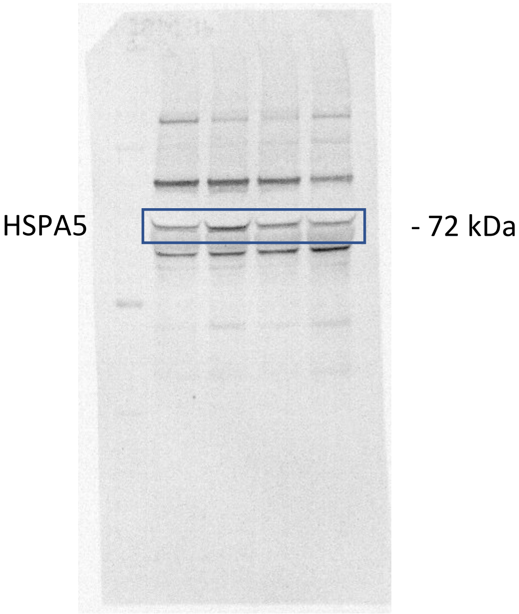

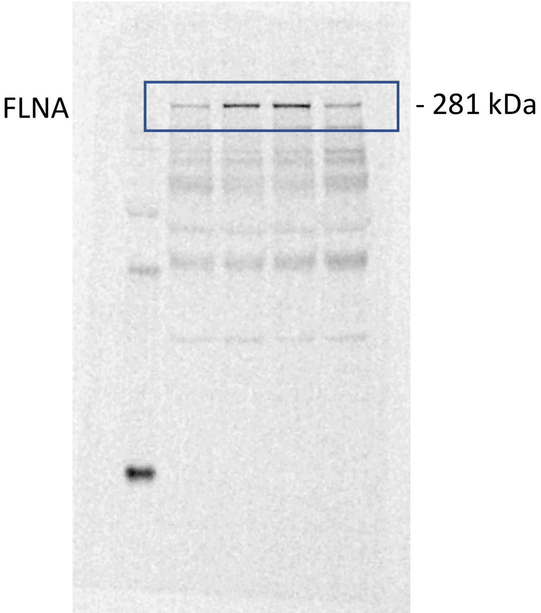

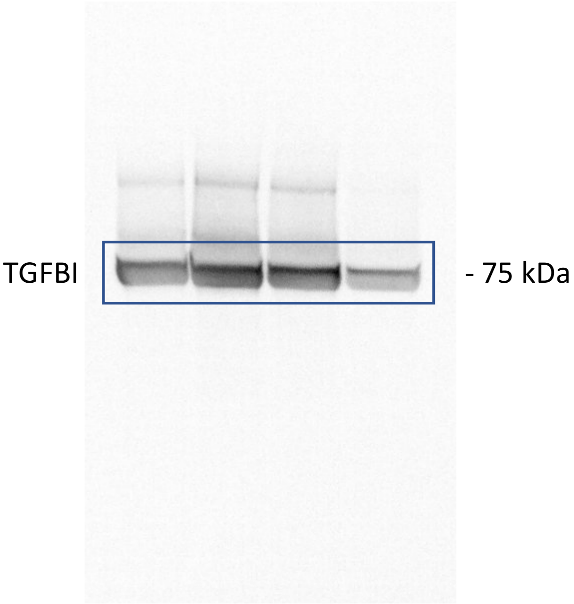

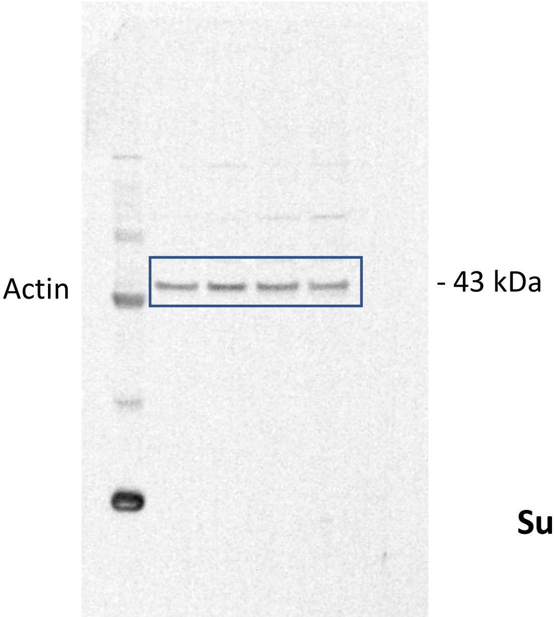

Supplementary Figure S8

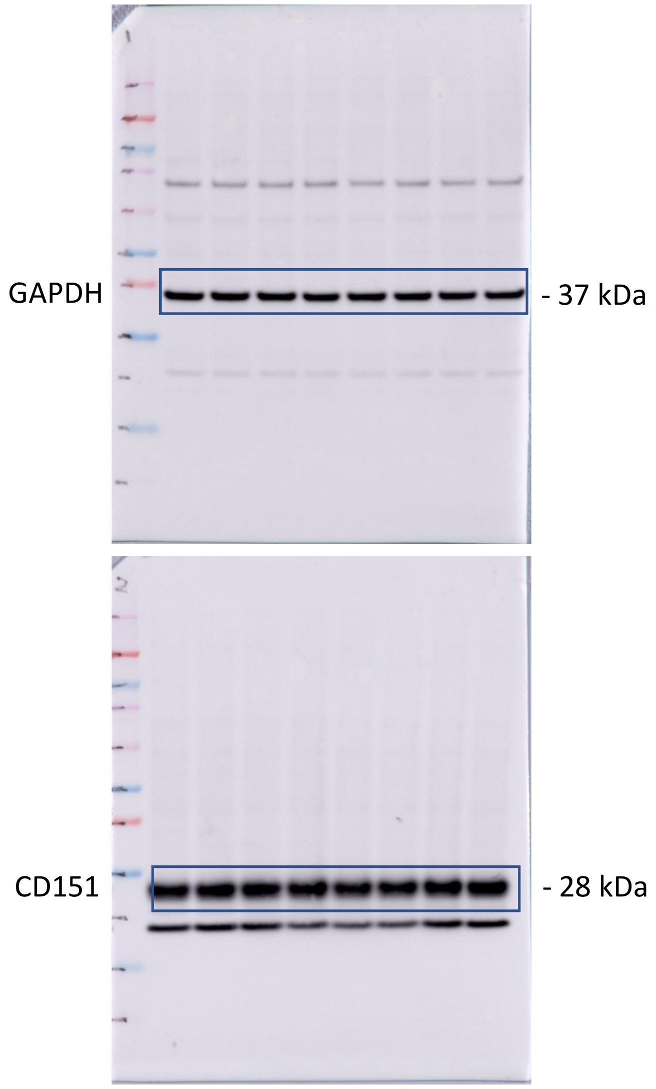

Supplementary Figure S9
